# Proximal ulna morphology in various pediatric population age groups: proximal ulna dorsal angulation (PUDA) and olecranon tip-to-apex (TTA) distance

**DOI:** 10.1007/s00276-023-03158-z

**Published:** 2023-05-13

**Authors:** Marcin Mostowy, Joanna Paul, Agata Majos, Charbel Mefleh, Marcin Piwnik, Piotr Kowalski, Szymon Kobielski, Stephen Choate, David Buzas, Adam Kwapisz

**Affiliations:** 1 Clinic of Orthopedics and Pediatric Orthopedics, Medical University of Lodz, str. Pomorska 251, 92-213 Lodz, Poland; 2 Department of Radiology, Medical University of Lodz, Lodz, Poland; 3 Radiology Department, Veteran’s Memorial Teaching Hospital in Lodz, Medical University of Lodz, Lodz, Poland; 4 Ochsner Health, Ochsner Sports Medicine Institute, 1201 S Clearview Parkway, Building B, Suite 104, Jefferson, New Orleans, LA 70121 USA

**Keywords:** Proximal ulna dorsal angulation, Tip-to-apex distance, Olecranon, Ulnar shaft, Fracture, Fixation, Child, Adolescent, Growth

## Abstract

**Purpose:**

To measure proximal ulna dorsal angulation (PUDA) and olecranon tip-to-apex distance (TTA) in pediatric population to aid surgeons with data for proximal ulna fractures fixation.

**Methods:**

Retrospective review of the hospital radiographic database. All elbow radiographs were identified and after implementing exclusion criteria, included were 95 patients aged 0–10; 53 patients aged 11–14; and 53 patients aged 15–18. PUDA was defined as the angle between lines placed on the “flat spot” of the olecranon and the dorsal edge of the ulnar shaft and TTA as the distance between the tip of the olecranon to the apex of angulation. Two evaluators performed measurements independently.

**Results:**

In age group 0–10, mean PUDA was 7.53°, range 3.8–13.7, 95% CI 7.16–7.91, while mean TTA was 22.04 mm, range 8.8–50.5, 95% CI 19.92–24.17. In age group 11–14, mean PUDA was 4.99°, range 2.5–9.3, 95% CI (4.61–5.37), while mean TTA was 37.41 mm, range 16.5–66.6, 95% CI (34.91–39.90). In age group 15–18, mean PUDA was 5.18°, range 2.9–8.1, 95% CI (4.75–5.61), while mean TTA was 43.79 mm, range 24.5–79.4, 95% CI (41.38–46.19). PUDA was negatively correlated with age (*r* = − 0.56, *p* < 0.001), while TTA was positively correlated with age (*r* = 0.77, *p* < 0.001). Reliability levels of 0.81–1 or 0.61–0.80 were achieved for most of intra- and inter-rater reliabilities besides two levels of 0.41–60 and one of 0.21–0.40.

**Conclusion:**

The main study finding is that in most cases mean age-group values may serve as a template for proximal ulna fixation. There are some cases in which X-ray of contralateral elbow may provide surgeon with a better template.

**Level of evidence:**

II.

## Introduction

Proximal ulna dorsal angulation (PUDA) is the intersection angle of lines drawn along the dorsal flat surface of the olecranon and the dorsal prominence of the ulnar shaft. This angle typically measures typically between 4.3 and 8.5° in adults [1, 5, 12, 15, 22, 27]. Olecranon tip-to-apex distance (TTA) is the distance measured on the line tangent to dorsal flat surface of the olecranon, between proximal tip of olecranon and place of proximal ulna angulation [6, 15]. TTA was reported to be between 47.0 mm and 86.3 mm in adults [2, 15, 22]. Due to radioulnar functional interplay, restoring native PUDA and TTA after a proximal ulna fracture is crucial for posttraumatic elbow function [2, 14, 16, 20]. Sandman et al. reported that five degrees of proximal ulna malreduction may result in radiohumeral joint subluxation [16]. What is more, disturbed proximal radio-ulnar joint may cause long-term sequelae such as growth disturbance of the radius or ulna [4, 9]. In case of severe malunion it is possible to perform corrective ulnar osteotomy to restore proper anatomical alignment, however, it requires another surgery, with all associated risks and burdens [21].

While multiple authors measured PUDA [1, 5, 12, 15, 22, 27] and TTA [2, 15, 22] in adults, due to authors knowledge PUDA and TTA were not described in various children and adolescents age groups. Therefore, the aim of the study was to measure PUDA and TTA in different children and adolescents age groups to aid surgeons with data for reduction and fixation of proximal ulna fractures.

## Materials and methods

The study was approved by institutional Ethical Committee, decision number RNN/230/19/KE from 9th April 2019. Due to retrospective-database nature of the study Ethical Committee did not demand collecting individual informed consent.

**Fig. 1:**
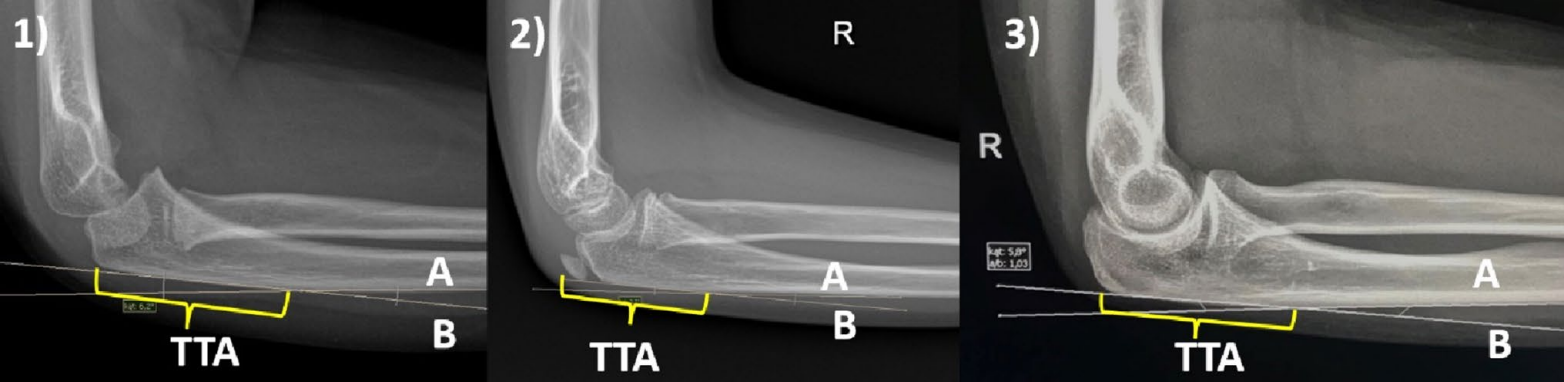
Lateral elbow projection; measuring Proximal Ulna Dorsal Angulation (PUDA) and olecranon Tip-To-Apex distance (TTA)

The study was designed as retrospective chart review of consecutive Radiology Department patients at the Central Teaching Hospital of the Medical University of Lodz, Poland. Sample size analysis was not performed, but all lateral elbow radiographs available in hospital database of patients 0–18 years old were identified. Exclusion criteria included radiographs with fractures around the elbow and radiographs with invalid lateral projection [19]. Valid lateral projection was defined as “the posterior supracondylar ridges of the humerus are superimposed, the radial tuberosity is oriented anteriorly, the radial head and coronoid process are partially superimposed, and the olecranon process is viewed in profile”, in agreement with Iyer et al. [8]. All radiographs were obtained using a digital imaging system (Siemens Healthcare). Commercially available imaging software (Exhibeon 2.7.21) was used to interpret the images. PUDA was defined as the intersection angle between tangent lines placed on the posterior “flat spot” of the olecranon and the dorsal prominence of the proximal ulnar shaft (Fig. 1). TTA was defined as the distance measured on the line tangent to dorsal flat surface of the olecranon, between proximal tip of olecranon and place of proximal ulna angulation (Fig. 1). The methods for PUDA and TTA measurements were the same as in the studies of Rouleau et al. and Han et al. [6, 15].

Examples of measurements from age groups: (1) 0–10 years old; (2) 11–14 years old; (3) 15–18 years old. Proximal Ulna Dorsal Angulation (PUDA) was defined as angle of intersection of lines A and B. Line A is tangent to the dorsal edge of the ulnar shaft and line B is tangent to the “flat spot” of the olecranon. Olecranon Tip-To-Apex distance (TTA) was defined as the distance measured on the line tangent to dorsal flat surface of the olecranon, between proximal tip of olecranon and place of proximal ulna angulation.

Two evaluators interpreted the radiographs independently. The first examination was performed as a trial run and the radiographs were reexamined by each evaluator at 1- and 2-week intervals after the first examination. The trial measurement was not included in outcome analysis. Radiographs were examined in a random order and results of previous measurements were blinded to avoid recall bias.

### Statistical analysis

The values of second and third examination were analyzed statistically using Statistica 13.1 software. Afterwards, intra-, and inter- reliability measurements were assessed using the Krippendorff alpha test. The following interpretation of reliabilities was utilized: poor < 0.20; fair 0.21–0.40; moderate 0.41–0.60; good 0.61–0.80; very good 0.81–1 [6, 10]. Final values of PUDA and TTA were calculated as mean with 95% CI from the means of two evaluators. The relationship between PUDA and gender was evaluated using either the student *t*- test or *U* Mann–Whitney test, accordingly to the data distribution [3, 13]. Normality of data distribution was evaluated by the Shapiro–Wilk test [18]. Statistical significance was set at *p* < 0.05.

## Results

**Fig. 2:**
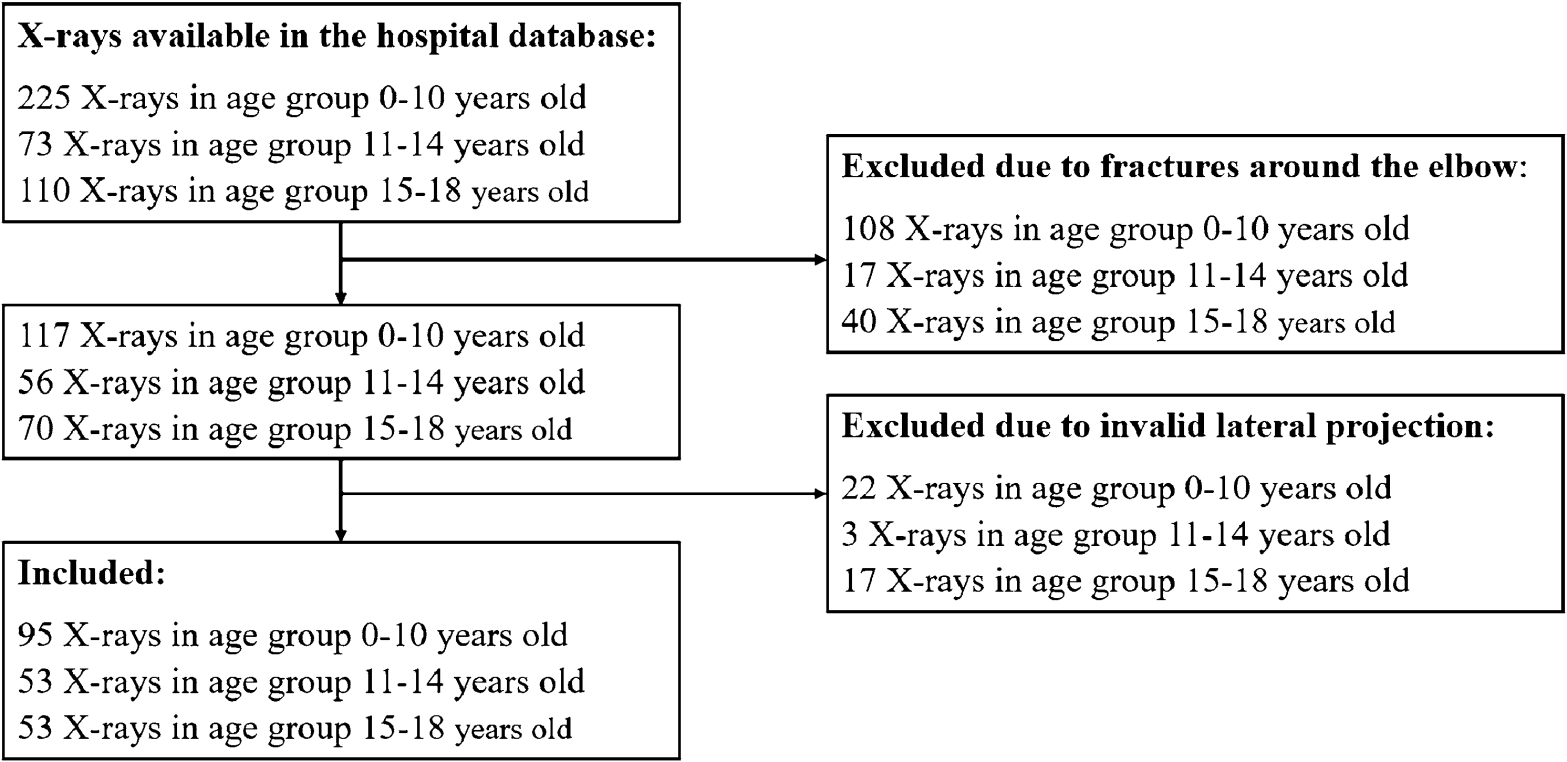
Flowchart of patients’ selection process

After the process of selection presented on the Flowchart in the Fig. 2, summarily 201 patients aged 0–18 (120 males and 81 females) were included. Details of demographics are presented in the Table 1.

**Table 1:** Demographics of included patients

Age group	Number of patients	Males	Females
0–10	95	52	43
11–14	53	33	20
15–18	53	35	18
All patients	201	120	81

**Fig. 3:**
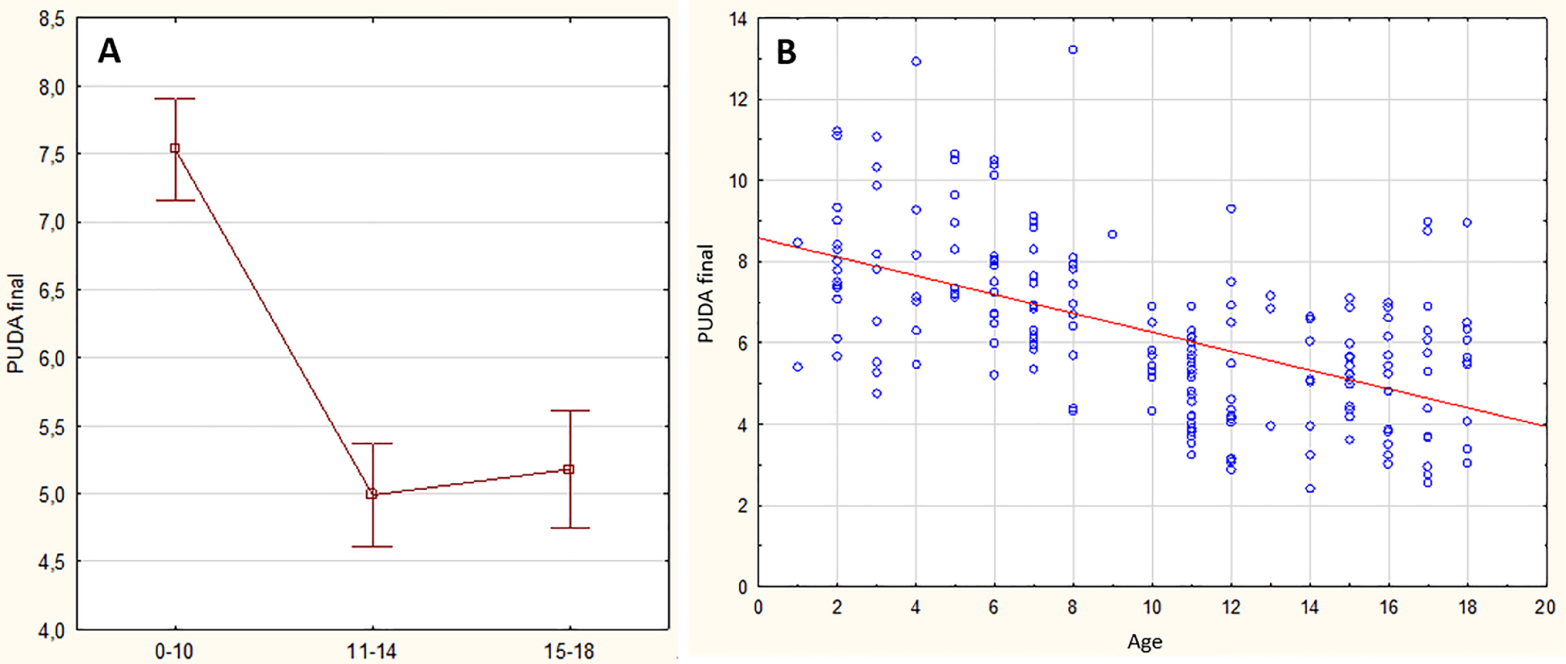
Final PUDA values in different age groups. PUDA—Proximal Ulna Dorsal Angulation; **A** graph presenting means and 95%CI of final PUDA values in age groups 0–10; 11–14; and 15–18. **B** graph presenting correlation between final PUDA value and age (*r* = − 0.56, *p* < 0.001)

Final values of PUDA and TTA measurements in different age groups are summarized in the Table 2, along with intra- and inter-rater reliabilities. PUDA was significantly higher in age group 0–10 compared with other age groups (*p* < 0.001) and it was negatively correlated with age (*r* = − 0.56, *p* < 0.001) (Fig. 3).

**Table 2:** Final values of proximal ulna dorsal angulation and olecranon Tip-To-Apex distance

Variable	Age group	Patients (*N*)	Mean	− 95% CI	+ 95% CI	1st observer intra-rater reliability	2nd observer intra-rater reliability	Inter-rater reliability
PUDA final	0-10	95	7.53	7.16	7.91	0.96	0.54	0.33
	11-14	53	4.99	4.61	5.37	0.97	0.80	0.50
	15-18	53	5.18	4.75	5.61	0.86	0.75	0.77
	All patients	201	6.24	5.96	6.53	N/A	N/A	N/A
TTA final	0-10	95	22.04	19.92	24.17	0.89	0.84	0.81
	11-14	53	37.41	34.91	39.90	0.75	0.79	0.88
	15-18	53	43.79	41.38	46.19	0.79	0.86	0.75
	All patients	201	31.83	29.94	33.71	N/A	N/A	N/A

*PUDA* proximal ulna dorsal angulation, *TTA* olecranon tip-to-apex distance, *CI* confidence interval, *N/A* not applicable

**Fig. 4:**
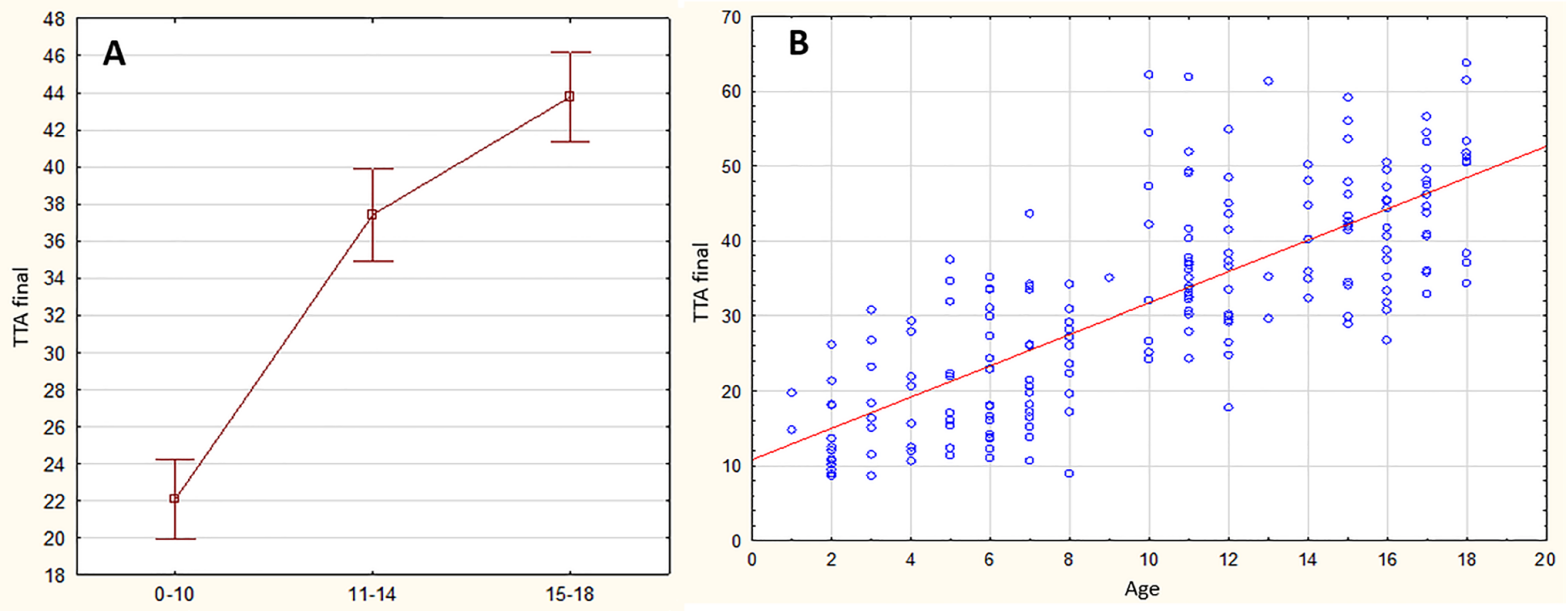
Final TTA values in different age groups. *TTA* olecranon Tip-To-Apex distance, **A** graph presenting means and 95% CI of final TTA values in age groups 0–10; 11–14; and 15–18. **B** graph presenting correlation between final TTA value and age (*r* = 0.77, *p* < 0.001)

TTA increased significantly in consecutive age groups (0–10 vs. 11–14, *p* < 0.001 and 11–14 vs. 15–18, *p* < 0.001) and it was positively correlated with age (*r* = 0.77, *p* < 0.001) (Fig. 4).

Mean final PUDA was higher in girls (6.65 ± 2.01) than in boys (5.67 ± 2.04, *p* = 0.021); however, this difference was not statistically significant when analyzed in age subgroups (Table 3).

**Table 3:** Associations between proximal ulna dorsal angulation and gender

Age group	Males			Females			
	Mean	SD	*n*	Mean	SD	*n*	*p*
0–10	7.41	1.91	52	7.68	1.75	43	0.57
11–14	4.70	1.24	33	5.46	1.52	20	0.30
15–18	5.01	1.41	35	5.51	1.79	18	0.24
All patients	5.67	2.04	120	6.65	2.01	81	**0.021**

Significant *p* value was bolded

*SD* standard deviation;

There were no significant differences in TTA between genders as well (Table 4).

**Table 4:** Associations between olecranon Tip-To-Apex distance and gender

Age group	Males			Females			
	Mean	SD	*n*	Mean	SD	*n*	*p*
0–10	22.17	11.71	52	21.88	8.77	43	0.89
11–14	38.32	8.81	33	35.91	9.45	20	0.35
15–18	44.95	8.17	35	41.51	9.55	18	0.18
All patients	33.26	14.13	120	29.71	12.44	81	0.069

*SD* standard deviation

## Discussion

This study provided original values of PUDA and TTA in various pediatric population age groups. Clinical importance of this study is that restoring preoperative PUDA and TTA is an important consideration in the operative treatment of proximal ulna fractures. Non-anatomic proximal ulna union can result in loss of range of motion, stiffness, radial head instability and early arthritis [14, 22]. Avoiding elbow arthritis is especially important due to the fact that treatment options are less effective and less predictable than those used for patients with i.e., hip or knee arthritis [11, 17, 23]. What is more, the cost and potential medical co-morbidity of additional surgery such as a corrective ulnar osteotomy to address a symptomatic malunion can be significant. This include increased risk for infection, suboptimal functional outcome, increased treatment cost, and additional time required for recovery [21]. Therefore, age-specific reduction should be performed [7, 22, 28]. Unfortunately, while as shown by 95%CIs, in most cases mean age-group values may serve as a template for proximal ulna fixation, there are some cases in which X-ray of contralateral elbow may provide surgeon with a better template.

It is difficult to directly compare the results of this study with the literature, as while there was a study assessing PUDA and TTA in children 0–12 years old [6], and there were many studies reporting these values in adults [1, 5, 12, 15, 22, 27], age-related change of PUDA and TTA in age range 0–18 years was not reported up to date. The results of this study remain in partial agreement with the study of Han et al., who reported significant negative correlation between PUDA and age in children 0–12 years old, however, they stated that no significant positive correlation between age and TTA was found (*r* = 0.254, *p* value not reported) [6]. However, in their study only subgroup 0–12 years old was assessed instead of full pediatric group 0–18 years old with age subgroups, as in this study [6]. Due to the fact that the study of Han et al. was the only available literature on pediatric PUDA and TTA, comparison with adult population was performed as well [6]. As to PUDA, in this study mean value measured in age group 0–10 years old (mean PUDA 7.53°) was higher than in adults, while in age groups 11–14 years old (mean PUDA 4.99°), and 15–18 years old (mean PUDA 5.18°), the results were comparable with the current published studies of adults. Rouleau et al. reported a mean PUDA value of 5.7 degrees in patients 18–80 years old, Yong et al. reported it to be 4.3° in patients 21–55 years old, Puchwein et al. measured a mean of 6.2° in patients ages 21–98 years old, Grechenig reported a mean PUDA of 4.5° in patients ages 59–98 years and Wang et al. reported a mean of 4.7° in patients aged 18–97 years old [12, 15, 24–27]. As to TTA, mean value in age groups 0–10 years old (mean TTA 22.04 mm) and 11–14 years old (mean TTA 37.41 mm), were lower than in adults. On the other hand, in age group 15–18 years old mean TTA was 43.79 mm, comparable with values reported by Rouleau et al. − 47 mm, Chapleau et al. − 47 mm, Wang et al. − 52 mm and Totlis et al. − 86.3 mm [2, 15, 22, 24, 25].

As to associations between PUDA, TTA and gender, the results of this study remain in partial agreement with the literature. In this study the mean PUDA value was higher in girls when all patients were accounted into analysis. However, no significant differences were observed in subgroup analysis. In the study of Han et al. concerning children 0–12 years old, no gender differences in any of analyzed morphological variables were found [6]. Authors commented on this suggesting that such result could be related to the fact that all included patients were prepubescent children [6]. In the literature concerning adult proximal ulna morphology, in the papers of Totlis et al. Wang et al. and Puchwein et al. there were no gender-associated differences of PUDA [12, 22, 24, 25]. Rouleau et al. reported no significant difference in PUDA between genders on the right side, however, on the left side PUDA was bigger in males (4.6 degrees for females vs 6.6 degrees for males) [15]. As to TTA, in this study it was higher in males in all age subgroups, however, statistical significance was not reached. In studies of Totlis et al. Wang et al. and Rouleau et al., TTA was reported to be bigger in males, while Chapleau et al. did not report TTA values for gender subgroups [2, 15, 22, 24, 25]. Many other characteristics of proximal ulna morphology were shown to be sex-dependent, for example length between the tip of olecranon and edge point (“height” of olecranon), varus angulation and olecranon length [12, 22]. Therefore, further reports regarding sex differences of proximal ulna morphology would be of interest in future research.

### Limitations of the study

This study holds some limitations. First, it was retrospective in design. However, all previous studies measuring PUDA or TTA were also performed either on cadavers or retrospectively [1, 5, 6, 12, 15, 22, 27]. Secondly, it is possible that number of participants for analysis of associations between gender, PUDA and TTA was too low. However, the primary aim of this article was to measure PUDA and TTA in different children and adolescents age groups, not necessarily to analyze gender associations. Third, while “good” or “very good” reliability levels were achieved for most of intra- and inter-rater reliabilities, two “moderate” and one “fair” reliability levels were achieved for 2nd observer intra-rater reliability in PUDA age group 0–10, inter-rater reliability for PUDA age group 11–14 and inter-rater reliability for PUDA age group 0–10, respectively. Lower reliability of measurements in younger age groups may be associated with lower bone mineralization than in older age groups. Achieved reliability values are similar to the other papers measuring PUDA or TTA, however, reliabilities were reported using different statistical outcomes [6, 15, 22]. Fourth limitation is that a priori sample size analysis was not performed. However, all available lateral elbow radiographs fulfilling study criteria were included. What is more, number of elbows analyzed in this study was higher than in many other studies assessing PUDA or TTA [1, 2, 5, 12]. Fifth limitation is that contralateral X-rays were not available for comparison in the database. This study provided original values of PUDA and TTA in various children and adolescents age groups. The authors believe this is useful information to aid surgeons with data for reduction and fixation of proximal ulna fractures through intramedullar pining or juxta cortical plating.

## Conclusion

This study provided original values of PUDA and TTA in various children and adolescents age groups. The main study finding is that while as shown by 95%CIs, in most cases mean age-group values may serve as a template for proximal ulna fixation, there are some cases in which X-ray of contralateral elbow may provide surgeon with a better template.

## Data Availability

The data is available on request from the corresponding author.
